# RETRACTION: Role of miR‐490‐3p in Blocking Bladder Cancer Growth Through Targeting the RNA‐Binding Protein PCBP2

**DOI:** 10.1002/kjm2.70009

**Published:** 2025-03-04

## Abstract

**RETRACTION:** ZhangC.‐M. , SongL.‐D. , WangJ.‐W. , YeH.‐B. , and ChenS. , “Role of miR‐490‐3p in Blocking Bladder Cancer Growth Through Targeting the RNA‐Binding Protein PCBP2,” Kaohsiung Journal of Medical Sciences 38, no. 1 (2022): 30–37. 10.1002/kjm2.12457.34622526 PMC11896500

The above article, published online on 07 October 2021, in Wiley Online Library (wileyonlinelibrary.com), and has been retracted by agreement between the journal Editor‐in‐Chief, Wan‐Long Chuang; Kaohsiung Medical University; and John Wiley and Sons Australia Ltd. The authors reported to the journal that further experiments with miR‐490‐3p mimic showed different results for cell viability than those that were presented in the published article. The authors further stated that these new results indicate that there are further errors in the results presented. Therefore, all parties agree that the article must be retracted. The authors did not respond to the notice regarding the retraction of their article.

**RETRACTION:** C.‐M. Zhang , L.‐D. Song , J.‐W. Wang , H.‐B. Ye , and S. Chen , “Role of miR‐490‐3p in Blocking Bladder Cancer Growth Through Targeting the RNA‐Binding Protein PCBP2,” Kaohsiung Journal of Medical Sciences 38, no. 1 (2022): 30–37. 10.1002/kjm2.12457.34622526 PMC11896500

The above article, published online on 07 October 2021, in Wiley Online Library (wileyonlinelibrary.com), and has been retracted by agreement between the journal Editor‐in‐Chief, Wan‐Long Chuang; Kaohsiung Medical University; and John Wiley and Sons Australia Ltd. The authors reported to the journal that further experiments with miR‐490‐3p mimic showed different results for cell viability than those that were presented in the published article. The authors further stated that these new results indicate that there are further errors in the results presented. Therefore, all parties agree that the article must be retracted. The authors did not respond to the notice regarding the retraction of their article.

